# Medicaid-Covered Peer Support Services Used by Enrollees With Opioid Use Disorder

**DOI:** 10.1001/jamanetworkopen.2024.20737

**Published:** 2024-07-09

**Authors:** Yuhua Bao, Hao Zhang, Kayla Hutchings, Rebecca Arden Harris, Tara Calderbank, Bruce R. Schackman

**Affiliations:** 1 Department of Population Health Sciences, Weill Cornell Medicine, New York, New York; 2 Department of Health Policy and Organization, The University of Alabama, Birmingham; 3 Department of Family Medicine and Community Health, Perelman School of Medicine, University of Pennsylvania, Philadelphia; 4 Penn Center for Mental Health, Perelman School of Medicine, University of Pennsylvania, Philadelphia

## Abstract

This cross-sectional study provides a multistate description of utilization of Medicaid-covered peer support services in 2019 by enrollees with opioid use disorder (OUD).

## Introduction

Peer support workers combine lived experience with formal training to support recovery of people with substance use disorder (SUD).^1^ They play an increasingly important role in addressing the opioid crisis.^2^ In the US, Medicaid covers approximately 40% of people with opioid use disorder (OUD).^3^ As of 2018, 37 Medicaid programs covered peer support services (PSS) for SUD.^4^ This study provides a multistate description of utilization of Medicaid-covered PSS in 2019.

## Methods

This cross-sectional study was approved by the institutional review board at Weill Cornell Medicine. Informed consent was waived because deidentified patient data were used, in accordance with 45 CFR §46. We followed the STROBE reporting guideline.

We synthesized existing information^4,5^ that identified 37 Medicaid programs that covered PSS for SUD in 2018, which were expected to be in implementation by 2019. Additional data collection was conducted on Medicaid coverage and payment rules (including payment codes if applicable) for PSS for SUD (eAppendix 1 in Supplement 1). We excluded 7 states from the original 37 because procedure codes did not exist or could not be identified to measure PSS for the Medicaid program and an additional 2 states because diagnostic or procedure codes for those states were considered unusable by the Medicaid Data Quality Atlas (eAppendix 1 in Supplement 1).^6^

We used 2019 data from the Transformed Medicaid Statistical Information System Analytical Files. The study population included Medicaid enrollees aged 18 to 64 years who had at least 1 diagnosis of OUD in any health care setting at any time in 2019. The number of days in 2019 for which an enrollee received Medicaid-covered PSS in either an individual or group setting were measured. We examined variation in the rate of PSS for at least 1 day across Medicaid programs (eAppendix 2 in Supplement 1). We tested differences in enrollee sex, race and ethnicity (collected by states at the time of Medicaid enrollment) (eAppendix 3 in Supplement 1), urban/rural location, and Medicare-Medicaid dual eligibility status by PSS use, with a 2-tailed χ^2^ test and clustering at the state level (*P* < .05 was considered statistically significant). For the 5 Medicaid programs with the largest number of enrollees using PSS, we examined the distribution of number of days receiving PSS. All statistical analyses were conducted with Stata MP version 18.0 (StataCorp) between October 2023 and April 2024.

## Results

**Figure 1.**
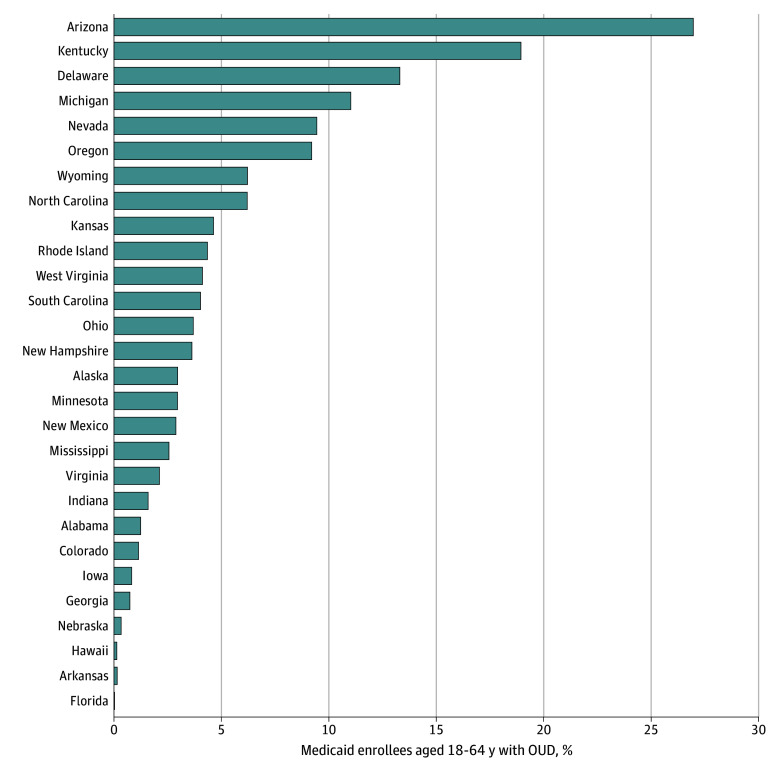
Percentage of Medicaid Enrollees Aged 18 to 64 Years With a Diagnosis of Opioid Use Disorder (OUD) Who Used Medicaid-Covered Peer Support Services for at Least 1 Day in 2019 Analysis included 28 states whose Medicaid programs covered peer support services for substance use disorder in 2018 with 1 or more known procedure codes. Denominator was all enrollees aged 18 to 64 years in 2019 with at least 1 diagnosis of OUD in any health care setting. A total of 617 066 enrollees were included in analysis. State-specific sample size ranged from 499 in Wyoming to 113 594 in Ohio.

The study sample included 617 066 individuals with OUD (288 821 [46.8%] male, 328 220 [53.2%] female; 35 060 [5.7%] Hispanic, 54 543 [8.8%] non-Hispanic Black, 435 882 [70.6%] non-Hispanic White, 91 581 [14.8%] with other race and ethnicity [Asian, American Indian or Alaska Native, Hawaiian or Pacific Islander, multiracial, missing]; and mean [SD] age, 38.4 [11.1] years). The proportion receiving at least 1 day of PSS ranged from 0.03% in Florida to 26.96% in Arizona, with a median of 3.30% (Figure 1). Users and nonusers of Medicaid-covered PSS did not differ significantly in terms of sex, race and ethnicity, or urban vs rural location. Users were less likely to be dually eligible for Medicare and Medicaid (5.8% [2757 out of 47 730]) than nonusers (10.7% [60 645 of 569 336]) (*P* = .007).

**Figure 2.**
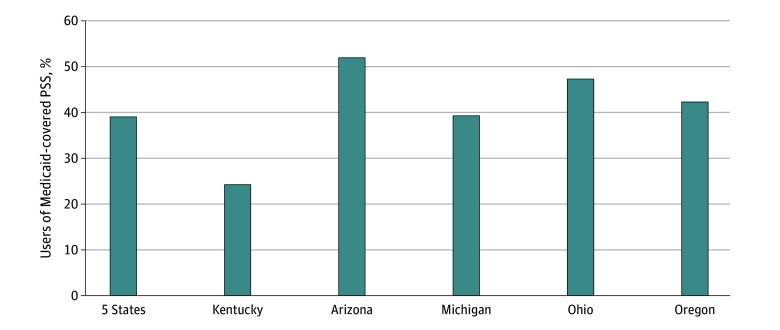
Percentages of Users of Medicaid-Covered Peer Support Services (PSS) Who Used the Services on 1 or 2 Days in 2019 Analysis included 5 states with the largest number of Medicaid enrollees aged 18 to 64 years who had a diagnosis of opioid use disorder in 2019 and who used Medicaid-covered peer support services for at least 1 day. Sizes of the denominators were 37 358 for all 5 states combined, 13 345 for Kentucky, 11 771 for Arizona, 5512 for Michigan, 4212 for Ohio, and 2518 for Oregon.

Among the 5 Medicaid programs (Kentucky, Arizona, Michigan, Ohio, and Oregon) with the largest number of enrollees who used PSS in 2019, the mean (SD) number of days receiving PSS was 10.4 (17.8) days (median [IQR], 4.0 [1.0-12.0] days; mode = 1.0); 39.0% of users received PSS on 1 or 2 days throughout the year, ranging from 24.2% in Kentucky to 52.0% in Arizona (Figure 2).

## Discussion

In 2019, overall use of Medicaid-covered PSS was infrequent among enrollees with OUD but varied substantially among the 28 states included in the study. Underutilization disproportionately affected dually eligible enrollees who, given the likely greater severity of their disability, could potentially benefit more from PSS. With only 1 to 2 days of Medicaid-covered PSS over an entire year for the largest proportion of users, PSS may not have been effectively utilized to support recovery. Study limitations include restriction to PSS identifiable with known procedure codes, likely variation in program maturity across states, and lack of considerations of other OUD treatment services. Future studies should seek to understand barriers to accessing and providing PSS to Medicaid enrollees and how Medicaid coverage and payment policies could be further developed to support effective delivery of PSS.

## Notes

Supplement 1. **eAppendix 1.** Inclusion of Medicaid Programs in Analysis and Procedure Codes Used to Measure Use of Medicaid-Covered Peer Support Services **eAppendix 2.** Derivation of Rate of Receiving PSS for at Least One Day **eAppendix 3**. Racial/Ethnic Categories of Medicaid Enrollees

Supplement 2.
Data Sharing Statement
