# Supplementary material for: Medicaid-Covered Peer Support Services Used by Enrollees With Opioid Use Disorder
Source: JAMA Netw Open. 2024 Jul 9;7(7):e2420737. doi: 10.1001/jamanetworkopen.2024.20737 (PMC11234232; doi:10.1001/jamanetworkopen.2024.20737)
Supplement: Supplement 1. — eAppendix 1. Inclusion of Medicaid Programs in Analysis and Procedure Codes Used to Measure Use of Medicaid-Covered Peer Support Services eAppendix 2. Derivation of Rate of Receiving PSS for at Least One Day eAppendix 3. Racial/Ethnic Categories of Medicaid Enrollees [file jamanetwopen-e2420737-s001.pdf]

Supplemental Online Content

Bao Y, Zhang H, Hutchings K, Harris RA, Calderbank T, Schackman BR. Medicaid-covered peer support services used by enrollees with opioid use disorder. *JAMA Netw Open.* 2024;7(7):e2420737. doi:10.1001/jamanetworkopen.2024.20737

eAppendix 1. Inclusion of Medicaid Programs in Analysis and Procedure Codes Used to Measure Use of Medicaid-Covered Peer Support Services

eAppendix 2. Derivation of Rate of Receiving PSS for at Least One Day

eAppendix 3. Racial/Ethnic Categories of Medicaid Enrollees

This supplemental material has been provided by the authors to give readers additional information about their work.

© 2024 Bao Y et al. *JAMA Network Open.*

eAppendix 1. Inclusion of Medicaid programs in analysis and procedure codes used to measure use of Medicaid-covered peer support services

In 2018, 37 Medicaid programs covered peer support services (PSS) for substance use disorder. From the 37 states, we excluded 4 states (MO, NJ, NY, PA) because Certified Community Behavioral Health Clinics (CCBHC) was the only or primary payment authority used by the Medicaid program to cover PSS as part of a comprehensive range of services. CCBHC is typically paid with a per diem or per month bundled payment. We were thus not able to tell if PSS was provided as part of CCBHC services. We further excluded 3 states (CA, IL, OK) because we could not identify the Healthcare Common Procedure Coding System (HCPCS) codes for PSS from original sources and examination by the research team of the state's data in TAF indicated that the state Medicaid program did not use H0038 – the most commonly used HCPCS code for PSS – in 2019. Finally, we excluded 2 additional states (UT and TN) because Medicaid Data Quality Atlas determined TAF data for these two states as having unusable procedure codes (UT) or unusable diagnostic codes (TN).

The following table provides the (non-CCBHC) Medicaid payment authority and HCPCS codes used to identify PSS for the 28 states included in the study.

State	Medicaid payment authority	HCPCS code(s) for PSS	Sources for HCPCS code(s)
AL	State plan rehabilitative services	H0038	https://medicaid.alabama.gov/content/Gated/7.6.1G_Archived_Manuals/7.6.1.G_PM_January_2020_Bookmarked.pdf
AK	State plan rehabilitative services	H0038	https://extranet-sp.dhss.alaska.gov/hcs/medicaidalaska/Provider/Sites/ArchivedFeeSchedule.html
AZ	State plan rehabilitative services	H0038	https://www.azahcccs.gov/PlansProviders/Downloads/FFSrates/Behavioral/FSBehavioralHealthOutpatientRates_01012018.pdf
AR	State plan rehabilitative services	H0038	https://humanservices.arkansas.gov/wp-content/uploads/180206_OBHS-fees.pdf<br><br>https://www.dfa.arkansas.gov/images/uploads/osp-anticipation-to-award/sp190054_AttachS.pdf
CO	1915(b)(3) waiver	H0038	https://spl.cde.state.co.us/artemis/hcpserials/hcp176internet/hcp176201807internet.pdf
DE	Section 1115 demonstration	H0038	https://medicaidpublications.dhss.delaware.gov/docs/DesktopModules/Bring2mind/DMX/API/Entries/Download?Command=Core_Download&EntryId=952&language=en-US&PortalId=0&TabId=94
FL	State plan rehabilitative services	H0038, H2015	https://ahca.myflorida.com/medicaid/rules/historical-medicaid-reimbursement-schedules
GA	State plan rehabilitative services	H0038, H0025	https://dbhdd.georgia.gov/provider-manuals-archive#fy18
HI	State plan rehabilitative services	H0038	https://www.thenationalcouncil.org/wp-content/uploads/2021/04/Hawaii_Billing_and_Financial_Worksheet.pdf

© 2024 Bao Y et al. *JAMA Network Open.*

IN	Section 1115 demonstration	H0038	https://www.in.gov/medicaid/providers/files/modules/behavioral-health-services.pdf
IA	1915(b)(3) waiver	H0038	https://provider.amerigroup.com/docs/gpp/IA_CAID__PF_BH_CoveredBenefits.pdf?v=202105182330
KS	State plan rehabilitative services	H0038	https://portal.kmap-state-ks.us/Documents/Provider/Provider%20Manuals/SUD_12122017_17200.3.pdf
KY	State plan rehabilitative services & Section 1115 demonstration	H0038	https://www.chfs.ky.gov/agencies/dms/DMSFeeRateSchedules/BHOutpatientNonFacilityFeeSchedule2020.pdf
MI	State plan rehabilitative services	H0038	https://midstatehealthnetwork.org/application/files/5615/5000/4108/Peer_Recovery_Services_-_Provider_Qualification_Changes.pdf
MN	State plan rehabilitative services	H0038	https://www.bluecrossmn.com/sites/default/files/DAM/2021-11/P11GA_17064718.pdf
MS	State plan rehabilitative services	H0038	https://www.dmh.ms.gov/wp-content/uploads/2020/02/DMH-Service-Provider-and-Billing-Manual-02252021.docx
NE	State plan rehabilitative services	H0038	https://dhhs.ne.gov/Medicaid%20Practitioner%20Fee%20Schedules/Mental%20Health%20and%20Substance%20Use%20July%201,%202019.pdf
NV	State plan rehabilitative services	H0038	https://www.medicaid.nv.gov/hcp/provider/Resources/SearchFeeSchedule/tabid/528/Default.aspx
NH	State plan rehabilitative services	H0038	https://www.nhhealthyfamilies.com/newsroom/Billing-of-Substance-Use-Disorder-Services.html
NM	Section 1115 demonstration	H0038	https://www.hsd.state.nm.us/wp-content/uploads/files/BH-POLICY-AND-BILLING-MASTER-.pdf
NC	1915(b)(3) waiver	H0038	https://medicaid.ncdhhs.gov/documents/files/8g-1/open
OH	State plan rehabilitative services	H0038	https://dam.assets.ohio.gov/image/upload/medicaid.ohio.gov/BH/provider/Manuals/Posted%20Manuals/BH-Manual-01302018.pdf
OR	State plan rehabilitative services	H0038	https://www.oregon.gov/oha/hsd/ohp/pages/fee-schedule.aspx
RI	Section 1115 demonstration	H0038	https://eohhs.ri.gov/sites/g/files/xkgbur226/files/2021-04/Peer%20Based%20Recovery%20Billing%20Manual%20v1.1.pdf
SC	State plan rehabilitative services	H0038	https://www.scdhhs.gov/sites/default/files/Copy%20of%20RBHS-DAODAS%20Provider%20-%20Rates%20eff%2012-1-2014%20web%20file.pdf
VA	State plan rehabilitative services	S9445, T1012, H0024, H0025	https://static1.squarespace.com/static/56d5ca187da24ffed7378b40/t/5e4e2ecc21989a778bc3db5f/1582182093508/OMCircle_ReferenceGuide_PeerSupport.pdf
WV	Section 1115 demonstration	H0038	https://dhhr.wv.gov/bms/Provider/Documents/Manuals/Chapter%20504%20Substance%20Use%20Disorder%20Services%20Manual%20FINALwithoutwatermark.pdf

© 2024 Bao Y et al. *JAMA Network Open.*

WY	State plan rehabilitative services	H0038	https://health.wyo.gov/wp-content/uploads/2018/04/Tribal-Manual0418-1.pdf

© 2024 Bao Y et al. *JAMA Network Open.*

eAppendix 2. Derivation of rate of receiving PSS for at least one day

Denominator – We identified Medicaid enrollees aged 18 to 64 in 2019 who had at least one inpatient or outpatient claim with a diagnosis of OUD. All diagnoses, primary or secondary, were considered. OUD was identified by ICD-10 diagnostic codes F11.x.

Numerator – Among enrollees identified as having an OUD diagnosis, we further determined whether the enrollee had at least one outpatient claim with a Healthcare Common Procedure Coding System (HCPCS) code indicating PSS covered by the state's Medicaid program (Table in Supplement A).

Rate of at least one day of PSS for each Medicaid program included in analysis = Numerator/Denominator

© 2024 Bao Y et al. *JAMA Network Open.*

eAppendix 3. Racial/ethnic categories of Medicaid enrollees

Medicaid-covered PSS users and non-users were compared and tested over several sociodemographic characteristics, including race/ethnicity. Racial/ethnic categories considered are: Hispanic, non-Hispanic Black, non-Hispanic White, and other. The “other” category includes Asian, Hawaii/Pacific Islander, American Indian or Alaska Native, multi-racial, and all other races.

© 2024 Bao Y et al. *JAMA Network Open.*
