# Supplementary material for: Medicaid-Covered Peer Support Services Used by Enrollees With Opioid Use Disorder
Source: JAMA Netw Open. 2024 Jul 9;7(7):e2420737. doi: 10.1001/jamanetworkopen.2024.20737 (PMC11234232; doi:10.1001/jamanetworkopen.2024.20737)
Supplement: Supplement 2. — Data Sharing Statement [file jamanetwopen-e2420737-s002.pdf]

Data Sharing Statement

Bao. Medicaid-Covered Peer Support Services Used by Enrollees With Opioid Use Disorder. *JAMA Netw Open*. Published July 09, 2024. doi:10.1001/jamanetworkopen.2024.20737

Data
Data available: No

Additional Information
Explanation for why data not available: The use of national Medicaid data in this study is governed by a DUA with the Centers for Medicare and Medicaid Services. Per the DUA, we are not able to share the data with the public.
